# Mobility of toxic elements in carbonate sediments from a mining area in Poland

**DOI:** 10.1007/s10311-014-0468-0

**Published:** 2014-04-23

**Authors:** Natalia Ospina-Alvarez, Łukasz Głaz, Krzysztof Dmowski, Beata Krasnodębska-Ostręga

**Affiliations:** 1 Faculty of Chemistry, University of Warsaw, Pasteura 1, 02-093 Warsaw, Poland; 2 Faculty of Biology, University of Warsaw, Żwirki i Wigury 101, 02-097 Warsaw, Poland

**Keywords:** Sequential extraction, BCR, Mining–tailing area, Thallium, Molybdenum, Poland

## Abstract

The Bolesław–Bukowno mining area in Poland is highly polluted by elements such as Zn, Pb, Cd and As. The reactivity and mobility of toxic elements such as Tl are poorly known. Here, we studied by sequential extraction the mobility of As, Cd, Co, Cr, Cu, Mn, Mo, Pb, Tl and Zn in sediments from two water reservoirs near Bukowno. Results show that available As, Co, Mn, Pb and Zn are found in carbonate minerals. Available Cd, Cu and Tl are found in sulphides and organic matter. The extractability of As, Cr, Mo and Tl was rather poor. By contrast, 85 % of total Cd, Pb and Zn was mobile. We discuss Tl and Mo association in carbonate sediments from ore deposits.

## Introduction

Bolesław–Bukowno mining area (Upper Silesia) is one of the most polluted areas in Poland. Approximately 2.2 and 0.1 million tons of Zn and Pb, respectively, have been smelted there since 1952 (Verner et al. 1996). Extensive sulphide ore mining, flotation treatment and smelting are the major sources of anthropogenic dispersion of metals in the vicinity of the zinc–lead smelters. The semi-liquid post-flotation wastes are deposited in large ponds located at the top of 25–30 m high heap. The heap is considered to be a main source of trace metals pollution in this area. The dried wastes can be easily spread by winds in form of dust in a range of several kilometres, due to vast surface and open character of the heap (Trafas 1996). Several elements such as As, Cd, Co, Cr, Cu, Mn, Mo, Pb, Tl and Zn are of high environmental interest due to their toxicity and therefore should be monitored, especially in polluted areas.

The post-flotation wastes contain dolomite, calcite, barite, sulphide and oxide minerals of iron, zinc and lead ores but also tailing metals such as Cd, Cu and Tl (Lis et al. 2003). These minerals are main components of the studied water reservoir bottom sediment. Trace metals exist mostly as carbonates or sulphides. Even small changes in water properties (pH and/or oxidation potential) can influence the leaching of these metals associated with carbonate and sulphide minerals. In the neighbourhood of the post-flotation waste heap (about 100 and 500 m far from its base), in the ground depression filled with seeping water, two ponds were formed.

Krasnodȩbska-Ostrȩga et al. (2005a) determined dissolved element fraction in surface water and total content in sediments for these water reservoirs, and their results pointed out that As, Cd, Cu, Mn, Pb, Zn, Se, Sb and Tl were available in bottom sediments in much higher concentrations than in water. Similar relation between metal content in water and sediments was found in comparable areas in Spain (Marqués et al. 2001) and China (Zhang et al. 2004).

After dissolved total quantification of elements in water and sediments, the next step of monitoring studies in this kind of reservoirs should be an estimation of mobility and fractionation of the studied elements. To estimate the mobile metal fraction available during natural processes in bottom sediments (for example, organic matter decomposition and oxidation of sulphide minerals and/or dissolution of carbonate minerals resulting in pH lowering of water being in equilibrium with the sediments), various reagents have been proposed. Considering the diversity of procedures and the lack of uniformity in different protocols, a sequential extraction scheme of the Standards, Measurement and Testing programme (SM&T, formerly BCR) was developed (Ure et al. 1993). This procedure have been accepted by other specialists and directly applied (Yuan et al. 2004; Zhang et al. 2004; Kowalska et al. 2002) or modified (Sutherland and Tack 2007; Prego et al. 2014; Kaplan et al. 2011). The acetic acid-available metal fraction is widely accepted as mobile fraction of trace metals in solid samples. The hydrogen peroxide-available fraction estimated basing on SM&T sequential extraction scheme is accepted as a metal fraction associated with organic matter and sulphide minerals.

The preliminary study of mineral components of the examined bottom sediments indicated that the sediments contain mostly carbonate minerals. Therefore, the aim of this study was to define the fractionation of As, Cd, Co, Cr, Cu, Mn, Mo, Pb, Tl and Zn in sediments rich in carbonates and poor in reducible minerals. The SM&T procedure of sequential extraction was modified taking in account the properties of investigated sediments.

## Experimental

### Sampling site

The sampling area is located in southern Poland, near zinc–lead mining area of Bolesław and smelting works in Bukowno (50.29°N, 19.49°W). The region belongs to the Silesian–Cracowian ore deposits of the ‘Mississippi Valley’ type, and the region is relatively rich in thallium (Lis et al. 2003). The sediment samples were collected from water reservoirs situated 100 m (Buk 2) and 500 m (Buk 3) away from the base on the heap in Bukowno. The ponds were formed in ground depressions filled with seeping waters. There are two main sources of trace metals: sediment–water exchange processes and the emission of metal-rich dust from the heap. The Buk 2 pond is regularly used during spring by frogs and toads as mating and breeding area, and Buk 3 pond is used by local inhabitants for fishing and recreation activities.

Bukowno–Bolesław district is characterised for the presence of minerals related to mining waste and ore smelting. The area is rich in meteoric minerals such as cohenite and graphite, sulphides of Fe, Cu, Pb, Zn (chalcopyrite, pyrrhotite, sphalerite, wüstite), spinels of Cr, Mg, Fe, Mn and Zn (chromite, magnetite), monoxides of Fe, Mg, Mn and Zn (wüstite, periclase) and silicate minerals (augite, omphacite, kirschsteinite). Mineral dressing waste contains mostly dolomite, limonite, Zn carbonates, Pb carbonates, sulphates, pyrite, and zinc and lead sulphides (Lis et al. 2012). Physical properties of the soil in the sampling area are detailed in Gruszecka and Wdowin (2013) (profiles I-A, II-A and III-0). In the first 10 cm of the soil, pH ranged from 6.6 to 8.0, organic matter from 30 to 80 % and reduction potencial from −21 to 21 mV.

### Reagents

Nitric acid (68 %, *d* = 1.42 g mL^−1^), hydrochloric acid (30 %, *d* = 1.15 g mL^−1^), hydrofluoric acid (40 %, *d* = 1.13 g mL^−1^), boric acid (4 %, *d* = 1.44 g mL^−1^) and acetic acid (96 %, *d* = 1.06 g mL^−1^) Suprapur^®^ (Merck, Germany) were used. Ammonium acetate and hydrogen peroxide were analytical grade (POCh Gliwice, Poland). Diethylene triamine pentaacetic acid, sodium acetate and sodium chloride were pro analysis grade (Merck). Multi-element standard solutions used for ICP MS calibration were prepared before measurements by dilution from a stock standard solution (SPEX CertiPrep^®^, Metuchen, NJ, USA). All solutions were prepared with Milli-Q water (Millipore, USA).

### Sediment digestion and sequential extraction

The sediment samples (three mixed sub-samples) were collected using PE vessels from a layer at a depth of 0–10 cm. The sediments with natural water content were open-air dried at the laboratory during 24 h and then oven-dried at 50 °C for 5 h. Dried materials were sieved through a 1-mm sieve. The material was homogenised by shaking. PE vessels with sediments were stored in darkness and ambient temperature until analysis.

Sediments were completely decomposed before determination by inductively coupled plasma mass spectrometry (ICP MS), following a three-step digestion using a microwave-system (Anton Paar, Austria) with high-pressure PTFE vessels. The chosen decomposition method was validated using certified reference materials (Schramel et al. 1996). Sediment samples intake was about 110 mg. Sediment samples were weighed into PTFE vessels, 2 mL of concentrated HNO_3_ and 1 mL of concentrated HCl were added to each vessel and screwed up tightly. The HPR rotor with vessels was placed on turntable of microwave oven, and microwave energy was applied in cycles: 5 min: 100 up to 500 W, 10 min: 500 W, 5 min: 500 up to 1,000 W and 45 min: 1,000 W. After 15 min of cooling down, the vessels were opened and 0.5 mL HF was added. The energy was applied again with the same cycles before described. After cooling, 5 ml of saturated H_3_BO_3_ solution was added and energy was applied as follows: 5 min from 100 up to 500 W and then 60 min at 600 W. After 15 min of cooling, the samples were dissolved in distilled water, quantitatively transferred into volumetric flasks of 30 mL and diluted to the mark.

For sequential extraction, one gram of sediment was transferred to an Erlenmeyer flask and 50 mL of CH_3_COOH (0.43 mol L^−l^) were added. The obtained mixture was shaken in horizontal position (16 h, 200 rpm) by means of a rotary shaker (Elpan 357). After shaking, extracts were immediately filtered through a filter paper previously rinsed with diluted nitric acid, followed by distilled water. The filtrates were collected in polyethylene tubes, and 0.1 mL of concentrated HNO_3_ was added.

Extraction with hydrogen peroxide, alone as well as in sequence with acetic acid, was done in quartz tubes. One-gram sample intake was leached with 10 mL of 30 % H_2_O_2_. The mixture was kept in room temperature for 1 h, until reaction nearly stopped. Then, the mixture was warmed up and kept for 1 h at 80–90 °C. Finally, 40 mL of CH_3_COONH_4_ (1 mol L^−1^) were added, and the solution was directly filtered. The extracts were stored at 4 °C until analysis. Blank extractions were carried out for each set of analysis using the same reagents as described above. All the blanks were handled and prepared following the same procedure used for the samples.

### Analytical procedure

Trace elements were determinate by means of ICP MS (SCIEX Elan 6000, Perkin Elmer, USA), according to the following parameters: sweep: 5; replicates: 5; dwell time: 0.1 s; ICP RF power: 1375 W; lens voltage: 11.3 W; nebulizer gas flow: 1.06 L min^−1^; plasma gas flow: 15.5 L min^−1^. The isotopes monitored were as follows: As 75, Cd 114, Co 59, Cr 52, Cu 63, Mn 55, Mo 95, Pb 208, Tl 205 and Zn 64. Total Quant™ software was used to automatically correct intensities for interference due to isobaric and molecular ions. Calibration curve method was applied as quantitative analysis approach.

Certified reference material (CRM: Trace elements in water, NIST-SRM 1643d,) was used to assess ICP MS measurements. Limit of detection (LOD) of the analytical procedure was calculated as a mean value plus 3 times the standard deviation of the blank ($\bar{x}$ ± 3 SD, *n* = 8). Reported values were always above detection limits.

In order to verify ICP MS determinations, Tl and Pb were analysed by means of anodic stripping voltammetry in some solutions after digestion and extraction according to Krasnodȩbska-Ostrȩga et al. (2005b) (data not shown). Tl was determined by means of differential pulse anodic stripping voltammetry (DP-ASV) using a µAutolab type II (ECO-Chemie, BV, The Netherlands) coupled with a 663VA Stand electrode system (3 mol L^−1^ Ag/AgCl reference electrode) (Metrohm, Switzerland), according to the following parameters, amplitude: 50 mV; scan rate: 10.5 MV s^−1^; deposition potential: −0.75 V vs 3 mol L^−1^ Ag/AgCl; deposition time: 2–5 min. 0.015 mol L^−1^ DTPA in 0.06 mol L^−1^ acetate solution (pH = 6.2) was used as supporting electrolyte. Determination of Pb was performed using subtractive anodic stripping voltammetry (S-ASV) with square wave mode (amplitude: 10 mV; step potential: 2.5 mV; frequency: 25 Hz; deposition potential: −0.80 V vs AgQRE; deposition time: 2–5 min). The solution containing 10 mmol L^−1^ NaCl and 10 mmol L^−1^ HNO_3_ was not deoxygenated. Silver rods (area: 1 cm^2^) dipped directly into solutions were used as working electrode and counter/reference electrode. S-ASV curves were obtained by automatic subtraction of oxidation currents recorded with and without accumulation.

SEM–EDS, scanning electron microscope (Zeiss LEO 435VP) equipped with EDS analyser (Roentec), was used for elementary analyses of main components in bottom sediments. Results of SEM–EDS analysis are presented as measured value ± uncertainty (Smith 2002).

## Results and discussion

The first step of the study was the determination of the main components of the collected sediments (Table 1). The obtained results show that Mn content was lower than 0.1 %, and content of Fe did not exceed 1.9 %. The ratio of Ca:Mg was close to 1, and sulphuric total content was not higher than 0.5 %. These results confirm that the samples were rich in dolomite and poor in sulphide and Fe–Mn minerals.

**Table 1:** Matrix of elements determination using SEM–EDS

Element/line		C (%) ± UNC	
		Buk 2 pond	Buk 3 pond
C	K-ser	8.05 ± 0.77	12.6 ± 1.1
Ca	K-alpha	1.72 ± 0.12	1.89 ± 0.19
O	K-ser	51.9 ± 4.3	57.8 ± 4.5
Fe	K-alpha	1.45 ± 0.18	1.58 ± 0.29
Mg	K-ser	1.48 ± 0.11	1.62 ± 0.16
Al	K-ser	2.85 ± 0.17	3.12 ± 0.14
Si	K-ser	29.8 ± 1.3	14.9 ± 0.6
S	K-ser	<0.3	0.52 ± 0.06

Data are presented as mean ± UNC

*SEM–EDS* scanning electron microscope with energy-dispersive detector, *UNC* uncertainty (calculated according to Smith 2002)

Mean element content in sediment samples after single and sequential extraction is presented in Table 2. Two-tailed *t* test were applied to (1) results by ICP MS and ASV (data not shown) and (2) obtained mean values and known values (CRM) (data not shown). Significant differences were not observed between both data sets (*p* < 0.05).

**Table 2:** Mean element concentration in sediment samples and extracts

	Buk 2-pond				Buk 3-pond			
	HAc	H_2_O_2_ − [Sq]	H_2_O_2_ − [S]	Total	HAc	H_2_O_2_ − [Sq]	H_2_O_2_ − [S]	Total
As	3.0 ± 0.2	0.21 ± 0.03	0.22 ± 0.08	22 ± 2	48 ± 2	0.84 ± 0.09	1.2 ± 0.03	320 ± 8
Cd	1.2 ± 0.3	0.17 ± 0.04	0.83 ± 0.07	1.5 ± 0.3	8.4 ± 0.7	90 ± 3	65 ± 2	110 ± 4
Co	1.3 ± 0.3	0.24 ± 0.09	0.82 ± 0.21	5.0 ± 3	2.4 ± 0.8	1.0 ± 0.4	3.1 ± 1.8	3.0 ± 2.1
Cr	1.3 ± 0.5	0.7 ± 0.2	0.7 ± 0.2	12.5 ± 0.5	1.5 ± 0.3	7.0 ± 3.0	8.4 ± 0.5	48 ± 1
Cu	64 ± 4	29 ± 1	15 ± 1	155 ± 43	2.6 ± 0.3	25 ± 1	28 ± 2	45 ± 5
Mn	129 ± 10	25 ± 3	113 ± 21	320 ± 26	760 ± 31	91 ± 4	608 ± 28	900 ± 32
Mo	0.13 ± 0.04	0.19 ± 0.06	0.20 ± 0.04	5.0 ± 0.2	0.17 ± 0.03	1.0 ± 0.2	0.63 ± 0.02	5.0 ± 0.4
Pb	357 ± 19	39 ± 2	37 ± 3	440 ± 37	1,491 ± 67	684 ± 30	48 ± 6	5,070 ± 260
Tl	0.14 ± 0.03	0.05 ± 0.02	0.12 ± 0.02	0.33 ± 0.05	1.2 ± 0.1	3.1 ± 0.1	4.0 ± 0.2	14 ± 3
Zn	1,106 ± 95	267 ± 10	593 ± 22	1,110 ± 110	15,110 ± 917	7,387 ± 188	10,332 ± 230	29,700 ± 210

Buk 2 and Buk 3 ponds correspond to the water reservoirs sampled in the study area of Bukowno–Bolesław (Southern Poland). Extractions were carried out with 30 % H_2_O_2_, alone as well as in sequence with 0.43 mol L^−l^ CH_3_COOH

[Sq]: extraction in sequence scheme; [S]: single extraction. Data are presented in mg kg^−1^ dry weight, as mean (*n* = 4) and standard deviations in parentheses

**Fig. 1:**
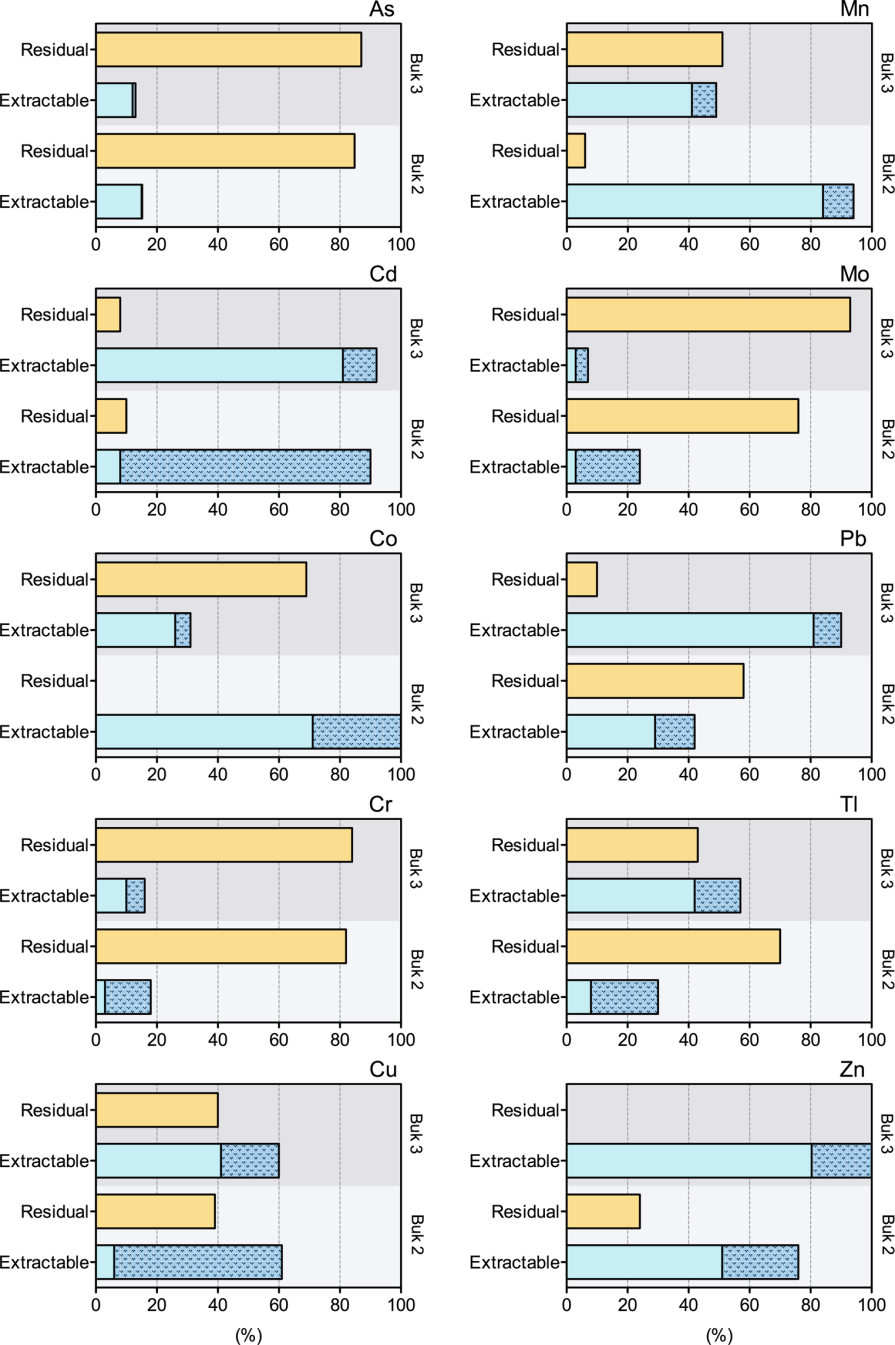
Relative proportion of extractability from bottom sediments of Bukowno–Bolesław mining/smelting area. *Light grey* area corresponds to the sampling site Buk 2 pond, and *dark grey area* to the Buk 3 pond. Extractable fraction corresponds to the sequential extraction carried out using hydrogen peroxide (*dark blue*, *dashed pattern*) and acetic acid (*light blue*, *without pattern*). Values are given in per cent (%) of total content. High extractability could be observed for Cd, Pb and Zn in contrast with the low extractability reached for As, Cr, and Mo, indicating different mechanism of mobilisation for these elements. The data were plotted using GraphPad Prism 5.0 for Mac OS-X (GraphPad Software Inc., 2007). (Color figure online)

According to the results obtained by SEM–EDS (Table 1), the fractionation study also indicated that bottom sediments contained rather dolomite. Acetic acid is commonly accepted reagent for estimation of easily exchangeable metal in sediments and soils (0.11 and 0.43 mol L^−l^ CH_3_COOH for sediments and soils, respectively) (Fletcher 1981; Relić et al. 2013). The almost complete dissolution of the sediment matrix was the first visible effect of the leaching with acetic acid solution. The residue contained mainly silicates and some brown matter. The large amount of Mn extracted with acetic acid could come from Mg/Ca carbonates; Mn^2+^ can substitute Mg^2+^ as well as Ca^2+^ in carbonate minerals, rather Mg in dolomite than Ca in calcite (Arunachalam et al. 1996). The extractability (relative mobility) of Zn and Co was large and reached even 90 % of their total content (Fig. 1). These elements are known to be associated with carbonates (Arunachalam et al. 1996; Zhang 2008). While calcite could be dissolved in relatively short time and at high pH, dolomite could be dissolved during longer time and lower pH (Zhang 2008). Therefore use of 0.43 mol L ^−1^ acetic acid was suitable for evaluation of mobile fraction in particular type of bottom sediment (Ure et al. 1993).

Unexpectedly high concentration of Pb, even 80 % of total content in acetic acid extracts (Fig. 1) indicated that the oxides minerals are not important for immobilisation of the metal. The published data pointed out that lead in presence of oxides minerals is mainly bound to this phase (Jakubowska et al. 2006; Krasnodȩbska-Ostrȩga et al. 2001; Sutherland and Tack 2007). The extractability discussed above and the results obtained using SEM–EDS revealed that metal fraction associated with reducible minerals is not important for immobilisation of lead in the studied bottom sediments.

The extraction efficiency for 0.1 mol L^−1^ hydroxylamine hydrochloride (NH_2_OH·HCl, pH 2) was also tested, and the results were close to the detection limits for most of studied elements. The relatively high extraction efficiency for Cr (6–15 %), Zn (25 %), Tl (15–22 %), Cu (20–55 %) and Cd (even 80 % for Buk2) obtained using hydrogen peroxide indicated that these elements were associated with the oxidable phase (Fig. 1). Studies of fractionation of solid samples by Kowalska et al. (2002) and Yuan et al. (2004), suggest that As, Cd, Cu, Cr, Ni and Pb are strongly bound to organic matter or sulphide minerals in the absence of Mn–Fe oxides phase (Zhang 2008); therefore, taking into account the results achieved with other extractants, the extraction with solution of hydroxylamine hydrochloride should not be considered in sediments with high content of carbonates.

It was noted a low extractability of As (Fig. 1), organic and sulphide fraction of As was less then 1 % of total arsenic content. On the other hand, the acetic acid-available arsenic fraction was relatively high—about 15 %. The extractability of As, Cr and Mo estimated basing on two steps stepwise extraction was lower than 20 % (Table 2). Besides Cd, Cu and Tl, the carbonate fraction was greater than the oxidable fraction in the proposed sequential extraction scheme (Table 2). Cd, Pb and Zn were in general the most mobile elements; even 90 % of total content of Cd was extracted in two steps of the proposed sequential extraction (Table 2, Buk 3 in contrast to Buk 2). Comparing the obtained results with the published ones about fractionation of elements based on BCR sequential extraction, (Guevara-Riba et al. 2004; Kowalska et al. 2002; Yuan et al. 2004; Zhang 2008), one might conclude that even small changes in pH and/or oxidising condition in soil environment could cause mobilisation of Cd, Pb and Zn. Apart from the stepwise extraction (acetic acid and hydrogen peroxide), single step extraction with hydrogen peroxide (pH 2) was also carried out. The reagent alone should be capable of simultaneous dissolution of carbonates and oxidising organic mater and sulphides.

In both sediment samples, it was noted that the sum of acetic acid and hydrogen peroxide fractions was always greater than release of the elements in single extraction with hydrogen peroxide solution (Table 2). The difference in leaching is especially noticeable for Pb and Zn. These results indicated that acetic acid solution did affect the trace element bound to organic phase. The hydrogen peroxide solution acidified with nitric acid up to pH 2 was not able to completely leach element fraction associated with acetic acid dissoluble phase of these bottom sediments. It was found for all studied elements that the total content and the mobile fraction (the fraction leached under SM&T extraction conditions) were higher in sediments collected from Buk 3 pond, than from Buk 2 pond. The extractability highly depends on composition of the sediment, and particle-size distribution and organic matter are relevant to the potential metal mobility (de Santiago-Martín et al. 2013); therefore, even small changes in the characteristics of the sediment could affect the extractability of the metals.

Previous studies indicated that the dissolved element fraction in surface water from Buk 3 pond was threefold to fivefold higher than in Buk 2 pond (Krasnodȩbska-Ostrȩga et al. 2005a). The extraction study showed that Co, Cd, Cu, Mn, Pb, Tl and Zn mainly bound to carbonates and sulphides (main components in the bottom sediments), are quite mobile. Basing on the fractionation study, it could be concluded that molybdenum extractability was rather poor and similar to As and Cr extractability from sediment matrix rich in carbonates and sulphides, in the absence of oxides and organic matter. The results of Cd and Tl leaching from these two sampling points differ in distance from post-flotation wastes heap, and the activity of live organisms could give some indirect evidence of their association with the sediment matrix. The immobilisation of trace metals depends on the presence of Mn–Fe oxides phase in solid matrix (Fletcher 1981; Krasnodȩbska-Ostrȩga et al. 2001), which was reflected by the poor immobilisation of Cd, Cu, Pb, Tl and Zn observed in the bottom sediment with low organic content. In these cases, the reducible and oxidable phase content is too low to effectively take part in the immobilisation of those metals (Jakubowska et al. 2006). It could suggest that the mechanism of Tl association with the sediment matrix rich in carbonates and sulphides in the absence of oxides and organic matter was similar to the mechanism of Pb. The results show that mobile fraction of Tl was associated with carbonate minerals.

The extractability of studied elements in sediment collected in Buk 3 pond beside higher total content was mostly equal (As, Cd, Cr and Cu) or even lower (Pb, Tl and Zn) than the extractability from Buk 2 sediment. It was probably correlated with the organic matter content, since if the content of total organic carbon (TOC) in sediment samples is high, the organic matter is mainly responsible for immobilisation of these elements (Kowalska et al. 2002).

The high mobility for Cd, Pb and Zn (≈85 %) here evaluated, opens up further avenues of research to be undertaken in this highly polluted area of Upper Silesia, such as evaluation of metal uptake by plants (phytoextraction, phytostabilisation, rhizofiltration, phytovolatilisation), and specific mobility studies of trace elements in order to assess its effect on the local wildlife.

## Conclusions

This study was conducted to evaluate the fractionation of trace elements in sediments rich in carbonates from mining and smelting areas. The results here obtained showed that carbonate minerals were responsible for immobilisation of As, Co, Mn, Pb and Zn, but the mobile fractions of Cd, Cu and Tl were associated with sulphides and organic matter phase. Fractions of As, Cr and Mo were strongly associated with the bottom sediments, and these elements would not be leached into water in relatively short time. It was found that Tl association mechanisms with sediments rich in carbonates and sulphides in the absence of oxides and organic matter tended to be similar to the Pb mechanism. Molybdenum extractability was poor and similar to the presented by As and Cr in the same kind of matrix. These findings provide new insight into Tl and Mo behaviour in complex matrices and could be specifically applied for understanding the role of essential (Mo) and non-essential (Tl) elements in plant metabolism.
